# An RNA-hydrolyzing recombinant minibody prevents both influenza A virus and coronavirus in co-infection models

**DOI:** 10.1038/s41598-024-52810-0

**Published:** 2024-04-11

**Authors:** Quynh Xuan Thi Luong, Phuong Thi Hoang, Yongjun Lee, Ramadhani Qurrota Ayun, Kyungho Na, Seonhyeon Park, Chengmin Lin, Phuong Thi Ho, Taek-Kyun Lee, Sukchan Lee

**Affiliations:** 1 Department of Integrative Biotechnology, Sungkyunkwan University, Suwon, 16419 Korea; 2 Ecological Risk Research Department, Korea Institute of Ocean Science & Technology, Geoje, 53201 Korea

**Keywords:** Biotechnology, Microbiology

## Abstract

With the lifting of COVID-19 non-pharmaceutical interventions, the resurgence of common viral respiratory infections was recorded in several countries worldwide. It facilitates viral co-infection, further burdens the already over-stretched healthcare systems. Racing to find co-infection-associated efficacy therapeutic agents need to be rapidly established. However, it has encountered numerous challenges that necessitate careful investigation. Here, we introduce a potential recombinant minibody-associated treatment, 3D8 single chain variable fragment (scFv), which has been developed as a broad-spectrum antiviral drug that acts via its nucleic acid catalytic and cell penetration abilities. In this research, we demonstrated that 3D8 scFv exerted antiviral activity simultaneously against both influenza A viruses (IAVs) and coronaviruses in three established co-infection models comprising two types of coronaviruses [*beta coronavirus*—human coronavirus OC43 (hCoV-OC43) and *alpha coronavirus*—porcine epidemic diarrhea virus (PEDV)] in Vero E6 cells, two IAVs [A/Puerto Rico/8/1934 H1N1 (H1N1/PR8) and A/X-31 (H3N2/X-31)] in MDCK cells, and a combination of coronavirus and IAV (hCoV-OC43 and adapted-H1N1) in Vero E6 cells by a statistically significant reduction in viral gene expression, proteins level, and approximately around 85%, 65%, and 80% of the progeny of ‘hCoV-OC43–PEDV’, ‘H1N1/PR8–H3N2/X-31’, and ‘hCoV-OC43–adapted-H1N1’, respectively, were decimated in the presence of 3D8 scFv. Taken together, we propose that 3D8 scFv is a promising broad-spectrum drug for treatment against RNA viruses in co-infection.

## Introduction

“Twindemic” or “Flurona” are terms coined during the COVID-19 pandemic that refer to concerns regarding simultaneous infection with both SARS-CoV-2 and influenza virus. During the 2020–2021 flu season, co-infection did not occur, possibly owing to the decreasing number of flu cases as a result of the large-scale implementation of non-pharmaceutical interventions against COVID-19^1,2^. However, along with Omicron surge and the resurgence of influenza virus in 2022 (cdc.gov/flu), an increase in flurona cases has been predicted, and it will undoubtedly put additional strain on healthcare providers already overloaded in response to COVID-19 pandemic. In recent studies, co-infection with influenza A virus (IAV) enhances the infectivity of SARS-COV-2, resulting in severe and prolonged pneumonia in hamsters and mice^3,4^. Since 2020, numerous articles have reported co-infection studies with diverse topics ranging from statistical reports to virus–virus and virus–host interactions, viral immunology, computational investigation of antiviral drugs against SARS-CoV-2, dengue virus, and chikungunya virus^5,6^, along with in vitro and in vivo studies of SARS-CoV-2, hCoV-OC43, IAVs, respiratory syncytial virus (RSV)^3,4,7–12^, and clinical studies^13,14^. Although no evidence of flurona causing the formation of hybrid viral particles exists, co-infection with strains or variants belonging to the same genus may easily occur, such as co-infection with two strains of influenza viruses or coronaviruses or two SARS-CoV-2 variants^15–17^. However, a 2022 report has described the formation of hybrid viral particles during co-infection with IAV and RSV^11^. In addition, to effectively mitigate and prevent the exacerbation of disease in co-infection, it is recommended to concurrently administer specific vaccines and antiviral drugs that target the coexisting viral pathogens within the host^18–20^. Nevertheless, drug resistance due to evolution of viruses and emergence of new viruses are challenges that constantly need to be addressed. Drug repurposing to treat other viruses has increasingly become attractive, although some obstacles remain to be overcome^18^.

Nowadays, the field of genetically engineered antibody technology has driven progress in the production of recombinant antibodies and antibody fragments, which play a vital role in research, diagnosis, and therapeutic strategies^21^. The single-chain variable fragment (scFv), which is one of the most popular types of engineered antibodies, is generated by fusing a variable heavy chain (VH) with a variable light chain (VL) via a flexible polypeptide linker^21,22^. In comparison to monoclonal antibodies, scFv exhibits several advantages, such as smaller size, enhanced tissue penetration, rapid circulation in the bloodstream, decreased retention in the kidney, low immunogenicity, high specificity, accomplished through bacterial expression systems, and facilitating large-scale manufacturing^21,23^. Numerous scFv variants have demonstrated substantial potential in diverse fields, including medical therapies and diagnostic applications. Several scFvs have been engineered to combat viral infections, such as those targeting chicken infectious bursal disease virus, human influenza virus H5N1, H1N1, and the phosphoprotein of Newcastle disease virus, PEDV^23–28^. Therefore, scFv represents a promising tool for the prevention and treatment of viral diseases. 3D8 scFv, a recombinant single-chain mini antibody (~ 28 kDa) was developed from autoimmune-prone MRL-lpr/lpr mice^29,30^. It has been shown to exert a broad-spectrum antiviral activity in vitro and in vivo against many types of DNA and RNA viruses, such as herpes simplex virus, pseudorabies virus ^31,32^, influenza viruses^33,34^, classical swine fever virus^35^, Newcastle disease virus^29,30,36^, and even the newly emerged SARS-CoV-2 virus^37^.

In this investigation, a greater multiplicity of infection (MOI) of viruses, higher concentration of 3D8 scFv, and later treatment timing, were utilized in comparison to prior single infection studies. We established that 3D8 scFv possessed antiviral effects against not only single infections of coronaviruses or IAVs but also co-infections of both viruses in three co-infection models: (1) coronaviruses (hCoV-OC43 and PEDV); (2) IAVs (H1N1/PR8 and H3N2/X-31); (3) coronavirus–influenza virus (hCoV-OC43 and adapted-H1N1). Our findings indicated that 3D8 scFv reduced viral genome levels, viral proteins, and progeny viruses by impeding the viral replication cycle, thus exerting antiviral activity in various cell line models utilizing an in vitro system.

## Results

### Inhibition of 3D8 scFv toward different viruses

**Figure 1:**
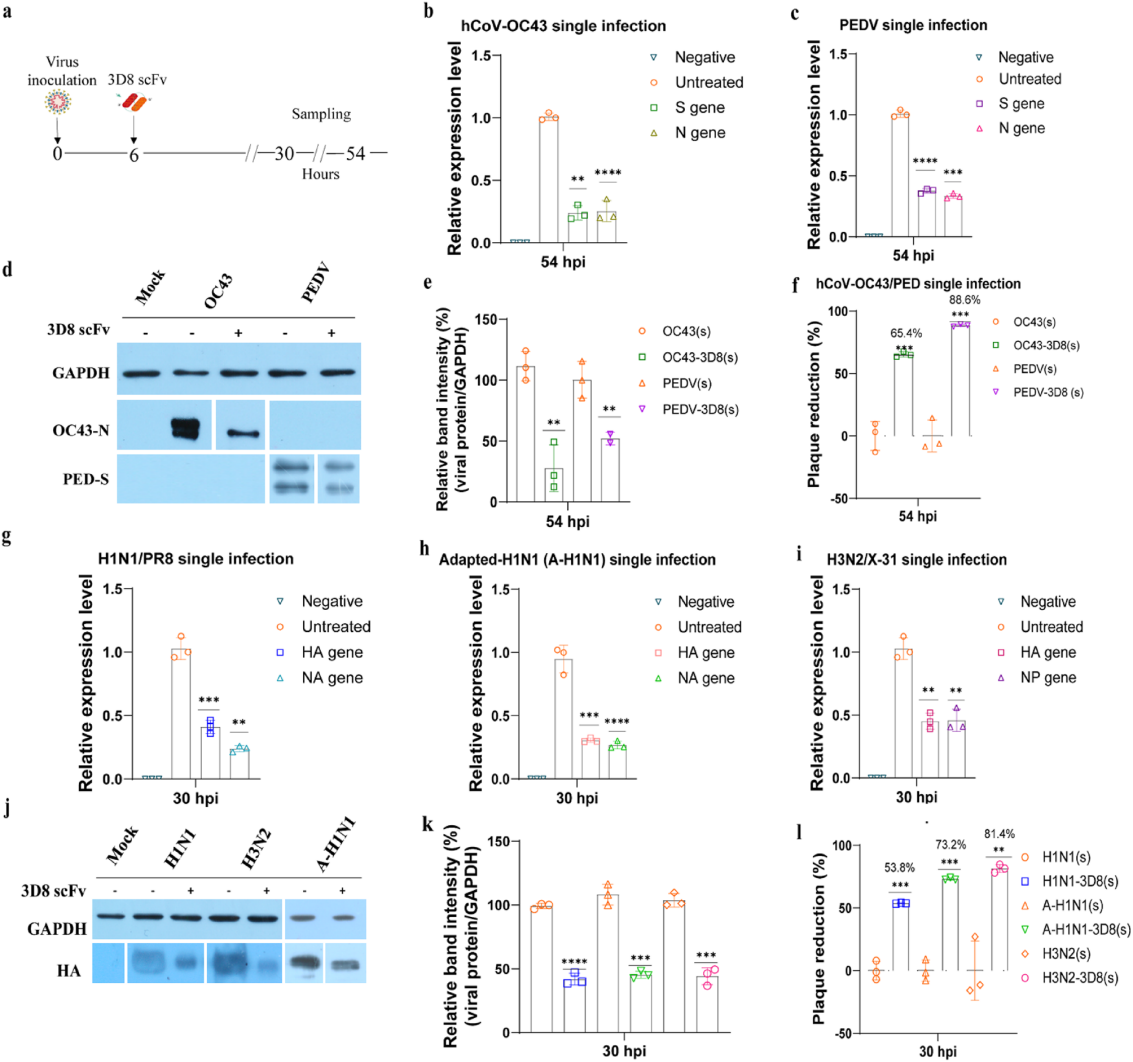
3D8 scFv protected cells from single infection with cytoplasmic or nuclear RNA viruses. (**a**) Scheme of the experimental procedure. Coronaviruses or IAVs were inoculated to Vero E6 or MDCK cells for 6 h. The cells and supernatants were collected at 24 or 48 h after treatment with 10 µM 3D8 scFv. One-step RT-qPCR was performed to determine the expression of the S and N genes of (**b**) hCoV-OC43 and (**c**) PEDV at 54 hpi. Simultaneously, the cells were lysed using RIPA buffer in order to check (**d**) the protein level of nucleoprotein (N) of hCoV-OC43 and spike protein (S) of PEDV via western blotting assay, (**e**) the percentage of band intensity was calculated based on normalizing viral protein intensity to GAPDH intensity. The supernatants from the 3D8-treated cells were harvested, and a plaque reduction assay was performed to observe the infectious viral titer, and percent plaque reduction of (**f**) coronaviruses hCoV-OC43 and PEDV. Similarly, HA and NA genes of (**g**) H1N1/PR8 and (**h**) adapted-H1N1 at 30 hpi, (**i**) HA and NP genes of H3N2/X-31 at 30 hpi were indicated by RT-qPCR. (**j**) The hemagglutinin (HA) protein levels of IAVs were tested, and (**k**) the percentage of band intensity was calculated based on normalizing viral protein intensity to GAPDH intensity. The percent plaque reduction of (**l**) influenza viruses H1N1/PR8, H3N2/X-31, and adapted-H1N1 was observed. All assays were in triplicates. The significant difference was determined by unpaired t-test (*P < 0.05, **P < 0.01, ***P < 0.001, ****P < 0.0001). The original western blots are presented in Fig. S4.

The broad-spectrum antiviral activity of 3D8 scFv against cytoplasmic and nuclear RNA viruses was investigated (Fig. 1a). Firstly, the expression of S and N genes of hCoV-OC43 and PEDV were strongly suppressed by up to 80% and 65%, respectively, in the 3D8 scFv-treated cells compared with those in untreated cells (Fig. 1b,c). Similarly, the viral protein level declined by 70% for hCoV-OC43 nucleoprotein and 50% for PEDV spike protein (Fig. 1d,e). Furthermore, the supernatants were harvested and subjected to plaque reduction assay, and the results showed that the progeny of PEDV and hCoV-OC43 was decreased by 89% and 66%, respectively (Fig. 1f and S3a).

Next, we investigated the antiviral effects of 3D8 scFv against H1N1/PR8, H3N2/X-31, and adapted-H1N1 in a single infection. The viral gene expression of IAVs declined by over 60% in 3D8-treated samples (Fig. 1 g,h,i), and similar results were observed for the viral protein level expression (Fig. 1j,k). Plaque reduction assay revealed that the progeny numbers of H3N2/X-31, adapted-H1N1, and H1N1/PR8 were decreased by 82%, 73%, and 54%, respectively (Fig. 1l and S3b). These results indicated that 3D8 scFv protected cells from virus in single infection models.

### Antiviral effects of 3D8 scFv against co-infection with coronaviruses or IAVs

**Figure 2:**
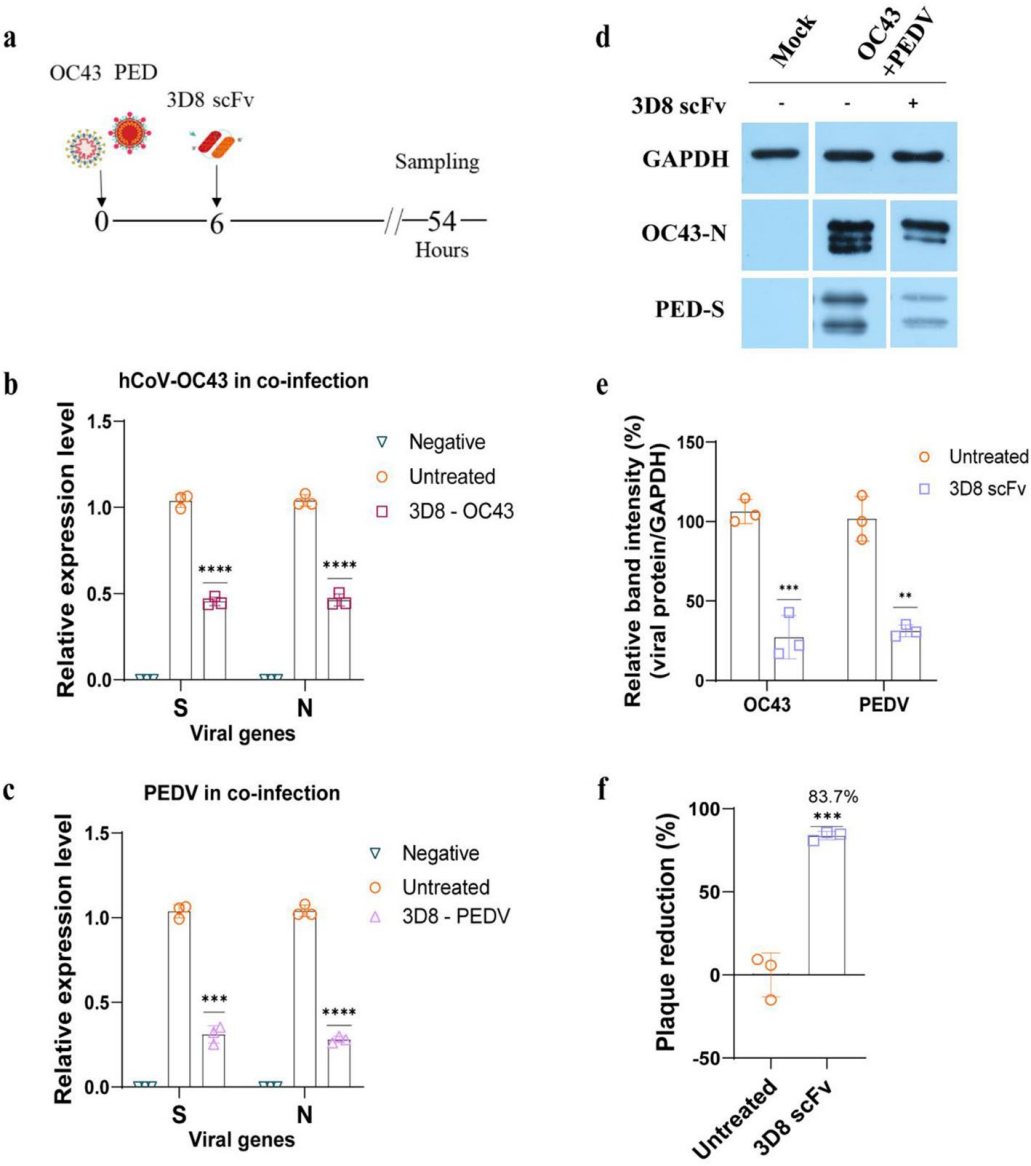
Antiviral activity of 3D8 scFv against co-infection with coronaviruses. (**a**) Diagram of procedure of co-infection with coronaviruses. 3D8 scFv (10 µM) was treated to Vero E6 cells co-infected with coronaviruses hCoV-OC43 (MOI 0.1) and PEDV (MOI 0.02). At 54 h after coronaviruses infection, the cells and supernatants were harvested to check S and N gene expression level of (**b**) hCoV-OC43 and (**c**) PEDV using one-step RT-qPCR, (**d**) determine viral protein level using western blotting, (**e**) the relative band intensity of hCoV-OC43 N protein and PEDV S protein was normalized to GAPDH intensity. The total viral titer of the two coronaviruses was detected using plaque reduction assay, the plaques were counted and (**f**) the percentage of plaque reduction was calculated. All assays were conducted in triplicates. Significant differences were determined using unpaired *t*-test (*P < 0.05, **P < 0.01, ***P < 0.001, ****P < 0.0001). The original western blots are presented in Fig. S4.

In the coronaviruses co-infection model, Vero E6 cells were challenged with both PEDV and hCoV-OC43 simultaneously (Fig. 2a). We assessed the inhibitory activity of 3D8 scFv against infection with two coronaviruses by evaluating the expression of the S and N genes of both viruses using western blotting and by assessing plaque reduction. Treatment with 3D8 scFv reduced the viral gene expression by 55% for hCoV-OC43 and 70% for PEDV (Fig. 2b,c and S2a). Only 25–30% of hCoV-OC43 nucleoprotein and PEDV spike protein were detected in 3D8 scFv-treated cells compared with those in the untreated cells (Fig. 2d,e). Furthermore, plaque reduction assay revealed that the generation of new virions in the co-infection model were markedly decreased by approximately 85% in the presence of 3D8 scFv (Fig. 2f and S3c).

**Figure 3:**
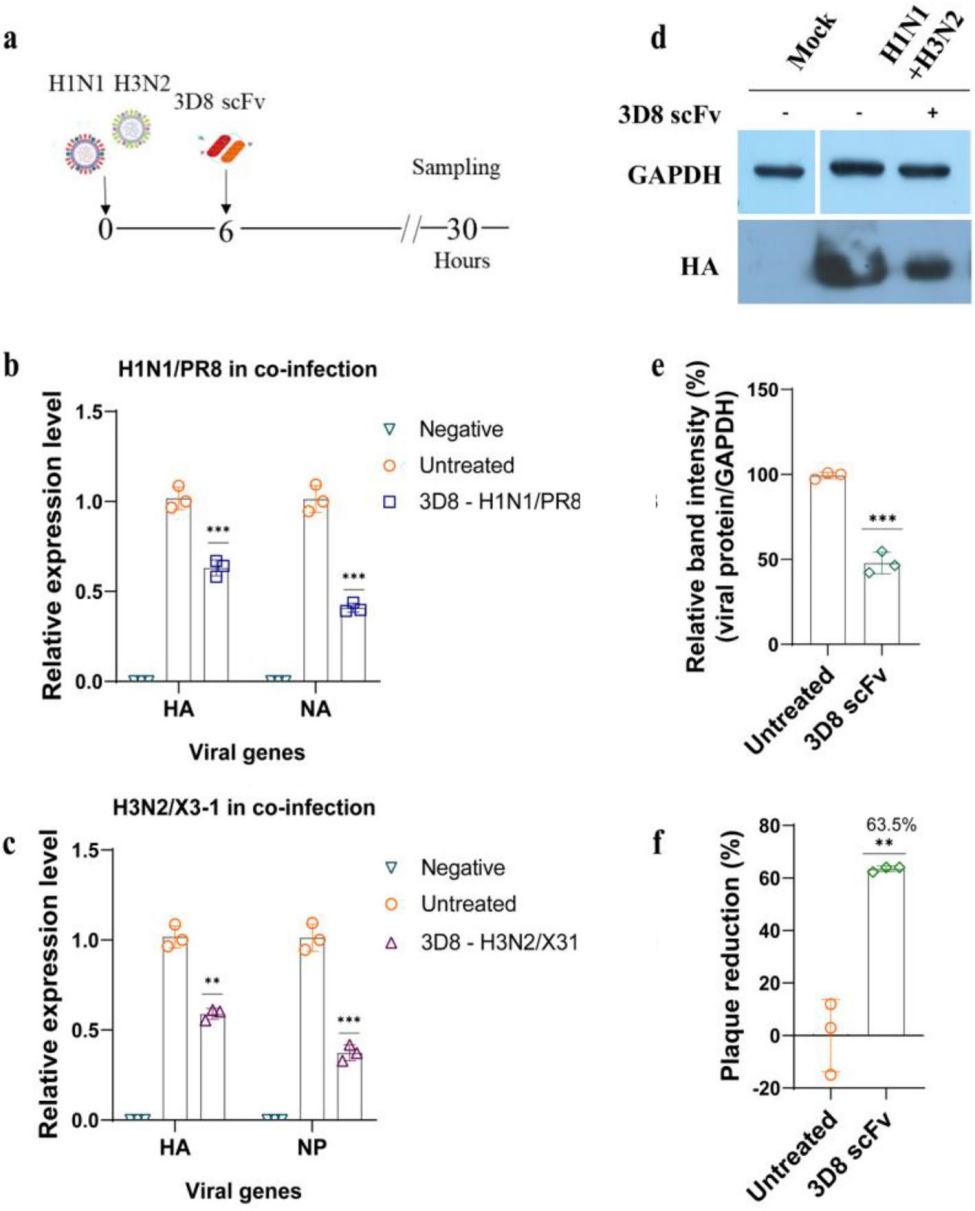
Antiviral effects of 3D8 scFv against co-infection with IAVs. (**a**) Diagram of procedure of co-infection with IAVs. MDCK cells were simultaneously inoculated with H1N1/PR8 (MOI 1) and H3N2/X-31 (MOI 2) at 37 °C. At 6 hpi, the cells were treated with 10 µM 3D8 scFv in MEM supplemented with 1 µg/mL TPCK, after which the cells were incubated for a total duration of 30 h. (**b**) HA and NA genes of H1N1/PR8 as well as the (**c**) HA and NP genes of H3N2/X-31 expression level in 3D8 scFv-treated and untreated cells were assessed using one-step RT-qPCR. In addition, (**d**) 3D8-treated cells were collected for western blotting assay using influenza HA primary antibody; (**e**) the viral protein expression was normalized to GAPDH expression. (**f**) Plaque reduction assay was conducted; the total progeny of IAVs in the co-infection model was quantified, and the percentage reduction was calculated. All assays were conducted in triplicates. Significant differences were determined using unpaired *t*-test (*P < 0.05, **P < 0.01, ***P < 0.001, ****P < 0.0001). The original western blots are presented in Fig S4.

To evaluate the ability of 3D8 scFv in blocking IAVs replication under co-infection, MDCK cells were simultaneously exposed to H1N1/PR8 and H3N2/X-31 for 6 h and then treated with 10 µM 3D8 scFv for 30 h at 37 °C, 5% CO_2_ (Fig. 3a). We found that the administration of 3D8 scFv inhibited viral gene expression following infection at high MOIs (H1N1/PR8, MOI 1; H3N2/X-31, MOI 2) (Fig. 3b,c and S2b). Western blotting showed consistent results, indicating that the expression of HA protein of both IAVs was greatly reduced by approximately 60% (Fig. 3d,e). The virus titer after 3D8 scFv treatment was detected in the supernatants, and the results showed a 65% decrease in total IAVs (Fig. 3f and S3d).

### 3D8 scFv protected Vero E6 cells against co-infection with two types of respiratory virus–coronavirus (hCoV-OC43) and influenza virus (adapted-H1N1)

**Figure 4:**
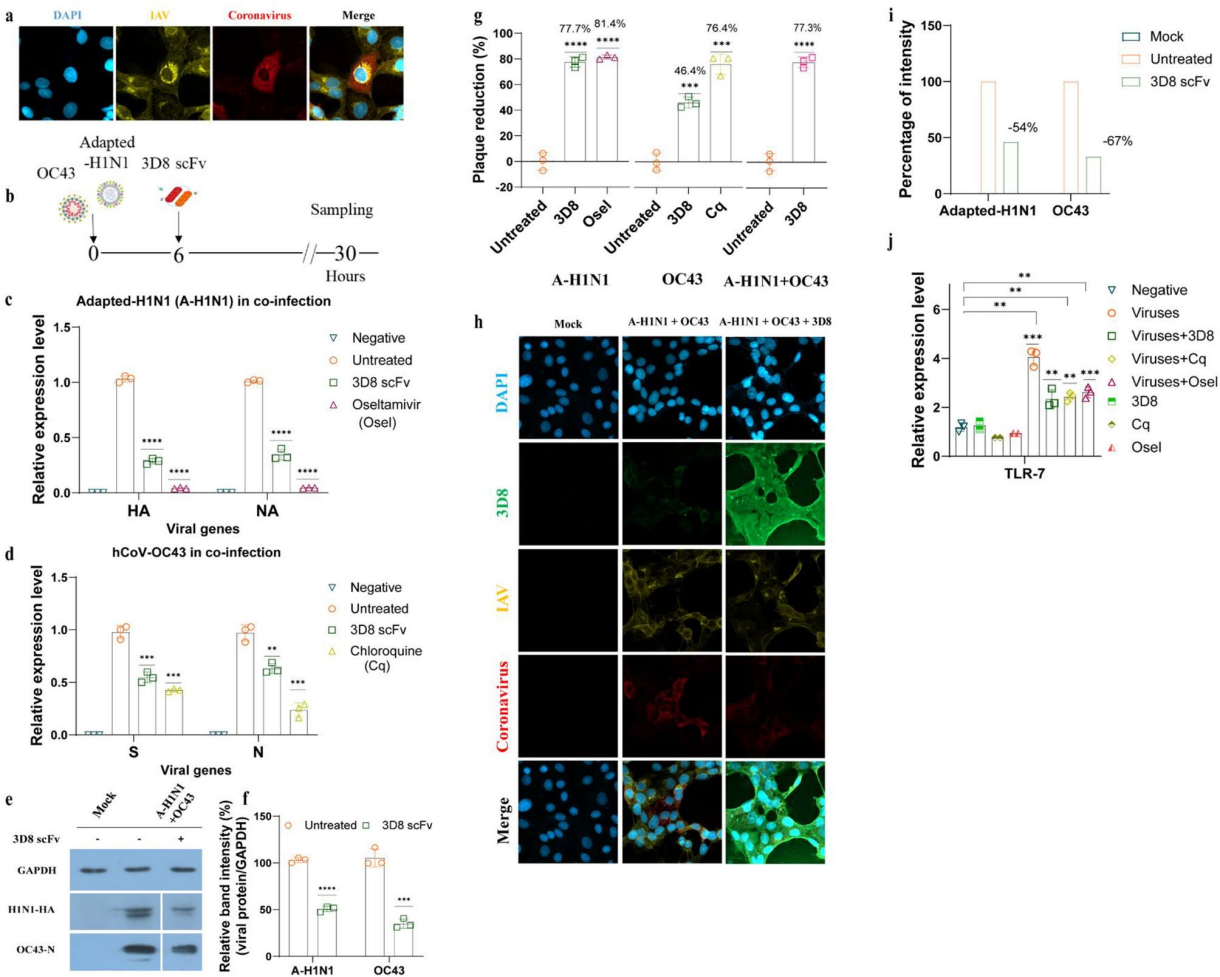
3D8 scFv suppressed co-infection with influenza virus and coronavirus. (**a**) Simultaneous infection of adapted-H1N1 (A-H1N1) and coronavirus (hCoV-OC43) was detected in infected Vero E6 cells using immunocytochemistry at 30 hpi. (**b**) Diagram of influenza virus (adapted-H1N1) and coronavirus (hCoV-OC43) co-infection procedure. Vero E6 cells were co-infected with adapted-H1N1 and hCoV-OC43 (MOI: 1 and 0.1, respectively). After 6 h of co-infection, the cells were treated with 10 µM of 3D8 scFv, oseltamivir (positive control), chloroquine (positive control), or DPBS (negative control) in DMEM supplemented with 1 µg/ml TPCK. (**c**) The HA and NA genes of adapted-H1N1 and (**d**) the S and N genes of hCoV-OC43 were quantified using one-step RT-qPCR. (**e**) The influenza HA protein and hCoV-OC43 nucleoprotein were examined via western blotting using the appropriate specific primary antibodies, in which (**f**) the relative band intensity of both viral proteins was normalized to GAPDH intensity. The supernatants were collected for progeny virus quantification using plaque reduction assay; plaques of adapted-H1N1 and hCoV-OC43 were separately counted, then (**g**) the plaque reductions for all treatments were combined in one graph to obtain the full view of plaque reduction under co-infection. (**h**) Antiviral activity of 3D8 scFv against adapted-H1N1 and hCoV-OC43 under co-infection in Vero E6 cells was measured using immunofluorescence, in which (**i**) the viral protein signal was converted to relative intensity percentages using CellProfiler 4.2.1, and the viral protein intensity was normalized to DAPI intensity. (**j**) *TLR7* expression level in treated and untreated cells. All assays were conducted in triplicates. Significant differences were determined via unpaired *t*-test (*P < 0.05, **P < 0.01, ***P < 0.001, ****P < 0.0001). The original western blots are presented in Fig. S4.

Our results revealed that 3D8 scFv protected host cells against co-infection with two cytoplasmic RNA viruses or two nuclear RNA viruses, which led us to a hypothesis that 3D8 scFv could block both hCoV-OC43 and adapted-H1N1 under co-infection. First, to establish a co-infection model comprising two different viruses in a single cell, the ability of both IAV and hCoV-OC43 to propagate in a single cell was confirmed (Fig. 4a). Next, Vero E6 cells were infected simultaneously with coronavirus and IAV as described in Fig. 4b. After 6 h of co-infection, 3D8 scFv was added at 10 µM to the cells. Our findings showed that the expression of HA and NA genes of adapted-H1N1 decreased by approximately 70%, whereas that of hCoV-OC43 S and N genes was reduced by 40% (initial MOI: 1 for adapted-H1N1 and 0.1 for hCoV-OC43) (Fig. 4c,d and S2c). Moreover, adapted-H1N1 HA protein and hCoV-OC43 nucleoprotein were detected in the cytoplasm of virus-infected cells, as assessed using western blotting. Approximately 50% of HA protein (adapted-H1N1) and 30% of N protein (hCoV-OC43) were detected in 3D8 scFv-treated cells (Fig. 4e,f). The number of progenies of adapted-H1N1 and hCoV-OC43 was approximately 3.7 × 10^7^ and 3.7 × 10^5^ progeny virions per milliliter, respectively, in Vero E6 cells without 3D8 scFv treatment (Fig. S3e). In contrast, in the presence of 3D8 scFv, the replication of adapted-H1N1 and hCoV-OC43 notably decreased by 80% and 50%, respectively (Fig. 4g). In comparison, oseltamivir-treated cells showed a similar percentage of IAV reduction, but chloroquine reduced hCoV-OC43 virion production by 77% (Fig. 4g). In addition, immunocytochemistry showed the intensity of coronavirus and IAV in the 3D8 scFv-treated cells was reduced by 67% and 54%, respectively, compared with that in untreated cells (Fig. 4h and i). Furthermore, we examined the expression of a host gene, *TLR7*, which is an innate immune receptor for viral ssRNA and plays an important role in host response to virus invasion^38,39^. *TLR-7* mRNA was upregulated under co-infection with adapted-H1N1 and hCoV-OC43 in Vero E6 cells with or without any treatments (Fig. 4j). However, 3D8 scFv-, chloroquine-, and oseltamivir-treated cells exhibited a notably decrease in *TLR-7* gene expression compared to the untreated cells (Fig. 4j). Considering that Vero E6 cells lack genes encoding type I interferons (IFNs), which play a role in the host innate immune system, our findings suggested that the antiviral activity of 3D8 scFv maybe not related to host antiviral immune responses during virus infection.

## Discussion

We recently reported that 3D8 scFv exhibited broad-spectrum antiviral activity against SARS-CoV-2 and other coronaviruses^37^. In addition, a prophylactic and therapeutic effect of 3D8 scFv against H1N1/pdm09 has been indicated^34^. It showed that 10 µM 3D8 scFv strongly reduced the gene expression of hCoV-OC43 and PEDV by approximately 100%, whereas 3 µM 3D8 scFv decreased IAV gene expression by approximately 50%^34,37^. As of the previous studies, our experiments showed similar results with reductions in viral gene levels, viral proteins, and progeny viruses. This finding suggested that 3D8 scFv exerted antiviral effects against two main RNA virus types, namely cytoplasmic and nuclear RNA viruses, which exhibit different replication cycles, in a single infection setting dependent on the MOI, protein concentration, and treatment timing after viral challenge. Besides, several studies have utilized single-chain variable fragment (scFv) antibodies to effectively neutralize the surface proteins of H1N1 and PEDV, thereby impeding viral replication in in vitro tests. Notably, recombinant engineered scFv NVLM10 and bivalent single-domain NVL2M10, which target the M2 protein, exhibited a remarkable 80%-90% inhibition of H1N1/PR8 infection^28^. *E. coli* expressing scFvs against PEDV showed a 94% reduction in viral foci compared to the positive infection control^40^. However, these findings highlight the significant potential of scFv in blocking the virus propagation by effectively neutralizing the target viral surface proteins as a prophylactic approach, our study revealed that 3D8 scFv has been a promising therapeutic approach as a broad-spectrum antiviral agent in viral disease prevention.

**Figure 5:**
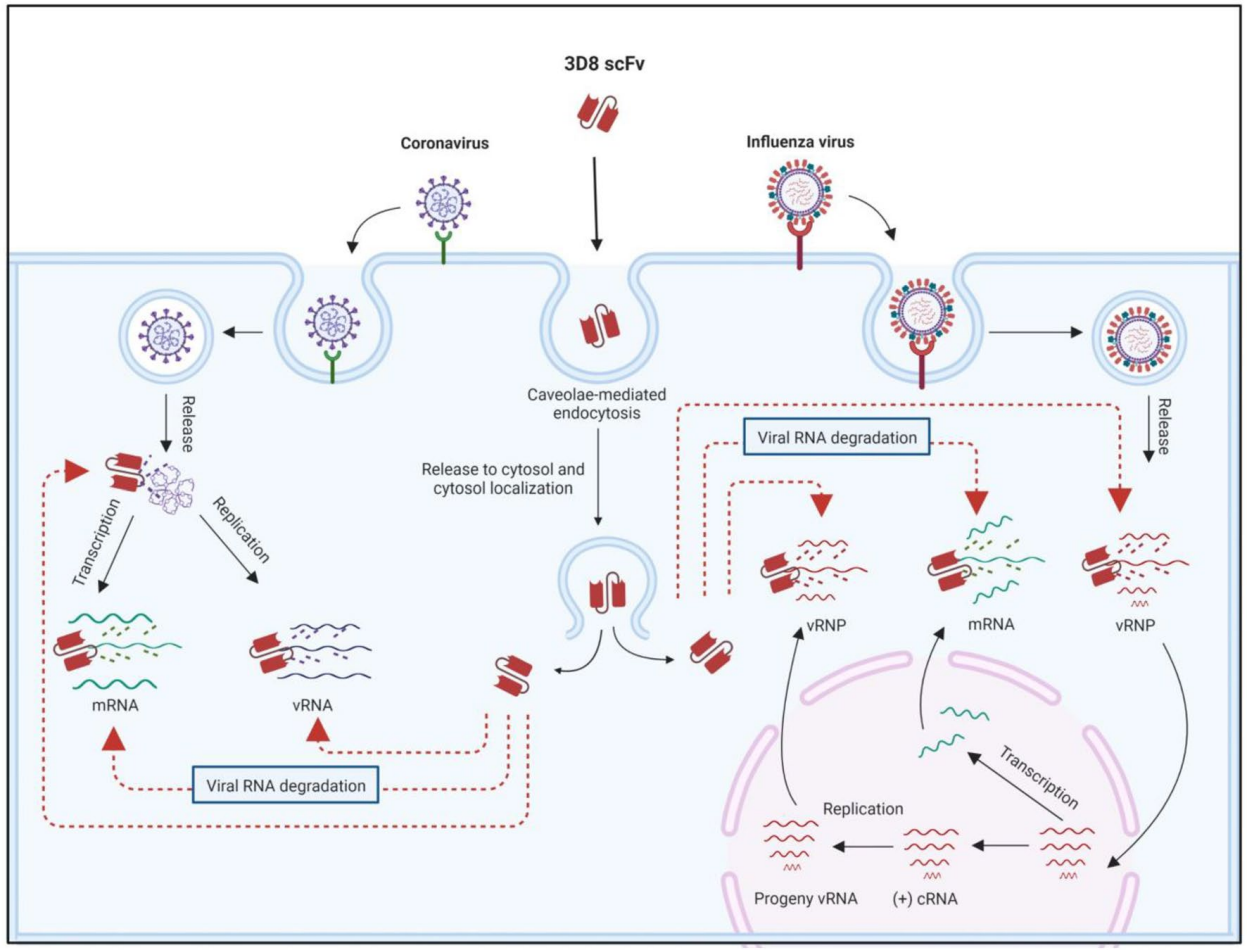
Proposed mode of action of 3D8 scFv against co-infection with coronavirus and influenza virus. Upon cell entry, 3D8 scFv localizes in the cytosol, where it binds to the viral nucleic acid (regardless of coronavirus or IAV) and viral mRNA and then degrades them via nuclease activity. As 3D8 scFv can hydrolyze all types of viral RNA in both viruses at different periods of viral life cycles, 3D8 scFv can inhibit viral propagation. Created with BioRender.com.

In an effort to mitigate the risk of co-infection and overcome drug resistance, 3D8 scFv was introduced as an antiviral drug capable of combating a broad range of viruses by directly targeting the viral genome to block viral replication. Influenza A virus is nuclear RNA virus, which means its replication occurs in the nucleus. 3D8 scFv has been shown to hydrolyze different types of IAV RNAs (vRNA, cRNA, and mRNA) at different infection stages: viral entry, viral protein synthesis, and viral exit to the cytoplasm^34^ (Fig. 5). hCoV-OC43 is a cytoplasmic RNA virus, and all of its replication steps occur in the cytoplasm, where 3D8 scFv is localized. Our study demonstrated that both the N and S genes of hCoV-OC43 were decreased by 3D8 scFv under both single infection and co-infection (Figs. 1b, 4d). We propose that 3D8 scFv targets coronavirus RNA in different stages of the viral life cycle. Thus, under co-infection with coronavirus and IAV, 3D8 scFv may act as a hunter, catching all types of viral RNA leading to reduction in viral proteins and new particle virions (Fig. 5). Currently, there is no approved antiviral drug available for the treatment of co-infection involving multiple viruses. Limited studies have been conducted on broad-spectrum antiviral agents targeting different viruses in both in vitro and in vivo settings. Recently, a study highlighted the potential of thapsigargin (TG), an inhibitor of the sarcoplasmic/endoplasmic reticulum Ca2 + ATPase pump, in inducing a host antiviral response that effectively inhibits co-infection with coronaviruses and IAVs^7,41^. Although the mechanisms of action and treatment approaches differ, both TG and 3D8 scFv demonstrated comparable efficacy. Notably, TG pretreatment exhibited a robust suppression of hCoV-OC43 progeny, reducing it by over 90% compared to the post-treatment approach using 3D8 scFv (Fig. 4g). Furthermore, 3D8 scFv demonstrated a substantial reduction in IAV progeny, inhibiting it by nearly 80% (Fig. 4g), surpassing the efficacy achieved by TG in the co-infection. Simultaneously, TG targets RSV and hCoV-OC43 at different stages of viral replication and also targets IAV at the post-translational stage^7^. We propose that 3D8 scFv targets the genomes of coronaviruses and IAVs at all stages of the viral life cycle. However, while TG was unable to prevent viral replication in cell lines that lack the type I IFN system, such as Vero E6 cells^7^, 3D8 scFv successfully exerted antiviral activity in both MDCK and Vero E6 cells in in vitro study.

Furthermore, *TLR7*, located on the X-chromosome, is an intracellular TLR that recognize single-stranded RNA and expressed in endosomes, lysosomes, and endolysosomes^38,39,42^. The primary role of TLR7 is conferring immunity against viral pathogens^38,42^. Upon recognizing viral ssRNA, TLR7 triggers several factors associated with the NF-kB pathway and inflammatory cytokine production and activates the expression of type I IFNs^38,39^. In our study, *TLR-7* expression was lower in the 3D8 scFv-treated cells than that in the untreated cells (Fig. 4j). Moreover, viral load declined after 3D8 scFv treatment (Fig. S3e), which may cause a decrease in the detection of viral ssRNA in endosomes and result in the downregulation of TLR-7 compared with that in untreated cells, leading to the reduction in type I IFNs and pro-inflammatory cytokines expression level. Additionally, the lack of genes encoding type I IFNs in Vero E6 cells validated the idea that 3D8 scFv was mainly responsible for the antiviral activity observed in this study.

Taken together, 3D8 scFv performed efficient antiviral activity against different strains, types, and subtypes of viruses in co-infection. With the notable efficacy of 3D8 scFv, which might directly target RNA viral genomes, it highlighted promising potential as a therapeutic drug against two different types of RNA virus not only in a single infection setting but also under co-infection. Furthermore, 3D8 scFv may have important implications for emerging or re-emerging RNA viruses-associated treatment in the future. We support the antiviral effect evaluation of 3D8 scFv against viral co-infection in vivo*.*

## Materials and methods

### Cell lines

Madin-Darby canine kidney (MDCK) (ATCC CCL-34) cells were maintained in complete Eagle’s minimal essential medium (MEM) (Hyclone, USA). Vero E6 cells (ATCC CRL-1586) were grown in complete Dulbecco’s modified Eagle’s medium (DMEM) (Hyclone, USA) at 37 °C and 5% CO_2_. The complete media were supplemented with 10% fetal bovine serum (Hyclone, USA) and 1% antibiotic–antimycotic (ThermoFisher Scientific, USA).

### Viruses

IAV strains A/Puerto Rico/8/1934 H1N1 and H3N2/A/X-31, which were kindly provided by Prof. Dae-Hyuk Kweon (Sungkyunkwan University, Republic of Korea), were propagated in the allantoic fluid of 9-day-old embryonated chicken eggs at 37 °C. The viruses were then collected and purified using sucrose gradient centrifugation. Vero E6 cell-adapted H1N1 (adapted-H1N1) was successfully generated from H1N1/PR8 as parental virus via virus adaptation method and propagated in Vero E6 cells. hCoV-OC43 (KBPV-VR-8) and PEDV (CV777), which were kindly provided by Novelgen research center (Suwon, Republic of Korea), were propagated in Vero E6 cells. All viral titers were determined by plaque assays.

### Preparation of 3D8 scFv

3D8 scFv gene was constructed in pIg20-3D8 plasmid (Fig. S1a), and expressed via the addition of 1 mM isopropyl 1-thiol-D-galactopyranoside (IPTG) to *Escherichia coli* BL21(DE3) pLysE strain in Luria–Bertani broth enriched with 100 g/mL ampicillin and 25 g/mL chloramphenicol for 18 h at 26 °C. The cell culture supernatant was obtained using centrifugation at 6000 rpm for 20 min at 4 °C and then filtered through a 0.22-mm filter. Next, 3D8 scFv was purified from the supernatant using an IgG Sepharose 6 fast-flow affinity column (GE Healthcare, USA). 3D8 scFv was then eluted with acetic acid (0.1 M, pH 3.4), and neutralized with a 0.1 volume of 1 M Tris–HCl (pH 9.0). The purity of the eluted protein was confirmed by SDS-PAGE with Coomassie-blue staining. All its key features were checked before being used for further experiments (Fig. S1b, c and d).

### Nucleic acid-hydrolyzing activity of 3D8 scFv

In order to confirm nucleic acid-hydrolyzing activity of 3D8 scFv, several types of substrates were used including pUC19 vector, ribosomal RNA, and viral RNA as double-stranded DNA, RNA substrates, respectively. The substrates (1 µg) were incubated with 0.5 µg of 3D8 scFv in 1X tris-buffered saline containing 0.1 mM MgCl_2_ at 37 °C for 0-15-30–-0 min, then analyzed using electrophoresis on a 1% agarose gel and stained with ethidium bromide. Total RNA was extracted from cell lines by using TRI reagent (MRC, USA), and viral RNA was synthesized using HiScribe T7 high yield RNA synthesis kit (New England Biolabs, USA) according to the manufacturing protocol.

### In vitro* antiviral activity of 3D8 scFv*

Three models of co-infection were established: (1) two coronaviruses [hCoV-OC43 (MOI 0.1) and PEDV (MOI 0.02)], (2) two IAVs [H1N1/PR8 (MOI 1) and H3N2/X-31(MOI 2)], and (3) coronavirus and influenza virus [hCoV-OC43 [MOI 0.1 and adapted-H1N1 (MOI 1)]. For model (2), the two IAVs were inoculated simultaneously to MDCK cells (1 × 10^5^ cells/well) seeded on a 24-well plate (SPL Life Sciences, Republic of Korea) for 1 h. After that, the infection medium was removed and serum-free medium containing 0.2% BSA and 1 µg/mL TPCK was then added to the virus-infected cells. At 6 h post-infection (hpi), 10 µM of 3D8 scFv was added to the cells, followed by incubation at 37 °C, 5% CO_2_ for 24 h. For model (1) and (3), Vero E6 cells (2 × 10^5^ cells/well) were seeded on a 24-well plate, washed twice with Dulbecco's phosphate-buffered saline (DPBS), and challenged with different pairs of viruses in serum-free media. Following 1 h of absorption, the infection medium was removed and replaced with complete DMEM for model (1) and DMEM including 0.2% BSA and 1 µg/mL TPCK for model (3). At 6 hpi, the cells were treated with 3D8 scFv. After that, supernatants and cells were collected at 54 hpi for model (1) and at 30 hpi for model (2) and (3), followed by storage at − 80 °C for further experiments. In which, cells were harvested using TRI reagent (MRC, USA) and RIPA buffer (Santa Cruz Biotechnology, USA) for RNA and protein extraction, respectively.

### RNA extraction and one-step quantitative reverse transcription PCR (RT-qPCR)

Total RNA was isolated using TRI reagent (MRC, USA), a final RNA concentration of 10 ng/μL was used. One-step RT-qPCR was performed using AccuPower GreenStar RT-qPCR Premix and Master mix (Bioneer, Republic of Korea) and Rotor-Gene Q system (Qiagen, German) with 50 ng of RNA template. Influenza virus genes (*HA* and *NA*), coronavirus genes (*N* and* S*), a toll-like receptor gene (*TLR-7*) were amplified using the primers listed in Table S1.

### Plaque assay and plaque reduction assay

MDCK and Vero E6 cells were seeded at 7 × 10^5^ cells/well in 6-well plates to 90–100% confluency. The supernatants harvested in the antiviral tests were serially diluted tenfold, and 1 mL of the diluted viral suspension was inoculated to DPBS-washed cells. Following 1 h of incubation, the cells were overlaid with DMEM containing 1% SeaPlaque agarose (Lonza, USA) with 1 μg/mL TPCK for model (2) and (3) or without TPCK for model (1). Plaque formation was observed for 3 and 4 days of incubation for influenza virus and coronavirus, respectively. Plaques were counted, and the percentage of plaque reduction was calculated.

### Immunoblot assay

Cells were lysed using RIPA buffer (Santa Cruz Biotechnology, USA) to extract the protein. Next, 20 μg of the protein was subjected to SDS-PAGE. Membranes after being transferred from gels were incubated with primary antibodies—monoclonal antibody to PEDV nucleoprotein protein (clone 3F12, 9191, Median Diagnostics, Republic of Korea), monoclonal antibody to hCoV-OC43 nucleoprotein (clone 542-7D, LS-C79764, LS-bio, USA), polyclonal rabbit anti-HA antibody to IAV (including H1N1 and H3N2) (PA5-349291, Invitrogen, USA), and polyclonal rabbit anti-GAPDH antibody (ab9485, Abcam, UK). After that, membranes were incubated with goat anti-mouse IgG-HRP conjugate (G-21040 Invitrogen, USA), and goat anti-rabbit IgG-HRP conjugate (A21020, Abbkine, USA). The membranes were added with Enhanced chemiluminescence (W3652-050, DawinBio, Republic of Korea) and exposed the film to observe the results. To analyze samples of viral co-infection, samples were divided into separated sets and then SDS-PAGEs were performed and transferred onto separated membranes. Each membrane was treated with different primary antibodies and secondary antibodies.

### Immunocytochemistry

Vero E6 cells (2 × 10^4^) were cultured in 8-well chamber slides. The hCoV-OC43 and adapted-H1N1 co-infection and 3D8 scFv treatment were performed as described above. The slides were fixed with cold-methanol and permeabilized with an Intracellular Staining Perm Wash Buffer (Biolegend, USA) for 15 min each. Following blocking with PBS with 0.1% tween 20 containing 1% BSA and glycine for 1 h, the cells were incubated with polyclonal rabbit anti-HA antibody (PA5-349291, Invitrogen, USA), monoclonal anti-coronavirus antibody (OC43 strain, clone 541-8F, MAB9012, Sigma-Aldrich, USA), and anti-3D8 antibody (humanized antibody, clone 1D7, Bioneer, Republic of Korea) at 1:1000 dilution for 24 h at 4 °C. After that, goat anti-human IgG Alexa fluor 488 (A-11013, Invitrogen, USA), donkey anti-rabbit IgG Alexa fluor 555 (ab150074, Abcam, UK), and goat anti-mouse IgG Alexa fluor 647 (ab1500115, Abcam, UK) were incubated for 1 h at 25 °C. The nucleus was stained with VECTASHIELD Antifade mounting medium containing DAPI (LSbio, USA) and visualized using a Zeiss LSM 900 confocal microscope (Zeiss, German). The viral protein signals were converted to relative intensity percentages using CellProfiler 4.2.1, and the viral protein intensity was normalized to DAPI intensity.

### Statistical analysis

All data were presented as the mean ± standard deviation (SD). GraphPad Prism version 8 (GraphPad Software, USA) was used to analyze the data. Two-tailed Student’s *t*-test was performed to compare the means of two groups. Differences of *P < 0.05, **P < 0.01, ***P < 0.001, or ****P < 0.0001 were considered significant.

### Supplementary Information

Supplementary Information.

## Data Availability

All datasets generated and/or analyzed during this study are included in this published article and its supplementary information files.
