# Supplementary material for: An RNA-hydrolyzing recombinant minibody prevents both influenza A virus and coronavirus in co-infection models
Source: Sci Rep. 2024 Apr 11;14:8472. doi: 10.1038/s41598-024-52810-0 (PMC11009316; doi:10.1038/s41598-024-52810-0)
Supplement: Supplementary file 1 — Supplementary Information. [file 41598_2024_52810_MOESM1_ESM.docx]

**An RNA-Hydrolyzing Recombinant Minibody Prevents Both Influenza A Virus and Coronavirus In Co-Infection Models**

Quynh Xuan Thi Luong^1^, Phuong Thi Hoang^1^, Yongjun Lee^1^, Ramadhani Qurrota Ayun^1^, Kyungho Na^1^, Seonhyeon Park^1^, Chengmin Lin^1^, Phuong Thi Ho^1^, Taek-kyun Lee ^2,*^, Sukchan Lee^1,*^

^1^ Department of Integrative Biotechnology, Sungkyunkwan University, Suwon 16419, Korea

^2^ Ecological Risk Research Department, Korea Institute of Ocean Science & Technology, Geoje 53201, Korea

*Correspondences:

Sukchan Lee
Email Address: [cell4u@skku.edu](mailto:cell4u@skku.edu)

Taek-kyun Lee

Email Address: [tklee@kiost.ac.kr](mailto:tklee@kiost.ac.kr)

**Supplementary tables**

**Table S1. Primers used in this study for RT-qPCR**

| **Primer name** | **Sequence (5’-3’)** | **Accession No.** |
| --- | --- | --- |
| **GAPDH** | F: GTCAGTGGTGGACCTGACCT  R: CCCTGTTGCTGTAGCCAAAT | XM_007967342.2 |
| **OC43-N** | F: CAACCAGGCTGATGTCAATA  R: AGCAGACCTTCCTGAGCCTTCAAT | MN310478.1 |
| **OC43-S** | F: GTACTGCTATTTCTGTTCAAACATGTG  R: GCTCTACGACTTCGTCTGTTTTTAGA | MN630560.1 |
| **PEDV-N** | F: ACAGGTCCAGATCTCCAAG  R: CCAAAGATTTAAGGGCATCCT | MW981424.1 |
| **PEDV-S** | F: ACGTCCCTTTACTTTCAATTCAC  R: TATACTTGGTACACACATCCAGA | MW826598.1 |
| **H1N1-HA** | F: AGTGCCCAAAATACGTCAGG  R: CAGTCCATCCCCCTTCAATA | NC_002017.1 |
| **H1N1-NA** | F: CCGGCAAAGTGATGTGTGTG  R: TTGTCACCGAAAACCCCACT | OM488264.1 |
| **H3N2-HA** | F: GAAAATGGTTGGGAGGGAATG  R: CATGGCTGCTTGAGTGCTTTT | DQ874876.1 |
| **H3N2-NP** | F: CAAAATCATGGCGTCTCAAGG  R: CGGTGCACATTTGGATGTAG | AB036779.1 |
| **TLR-7** | F: CTAAAATTGGCTCCGTGGAA  R: AAGAGGGGGCAGTGATAAA | XM_007991035.2 |

**Supplementary figures**

**Fig S1. Key characteristics of 3D8 scFv. (A)** 3D8 scFv expression plasmid. **(B)** The nucleic acid-hydrolyzing activity was exhibited through double-stranded DNA, ribosome RNA degradation, and **(C)** viral RNA degradation. **(D)** Cell penetration activity in Vero E6 cell line.

**Fig S2. Viral gene reduction was indicated by the inhibitory activity graph**. Inhibitory activity of 3D8 scFv against co-infection with **(A)** two coronaviruses, **(B)** two IAVs, and **(C)** IAV and coronavirus (based on viral gene expression).

**D**

**C**

**B**

**A**

**PEDV**

**Untreated**

**3D8 scFv**

**hCoV-OC43**

**10^-2^**

**10^-2^**

**H3N2**

**H1N1**

**10^-6^**

**10^-4^**

**Adapted-H1N1**

**10^-5^**

**Untreated**

**3D8 scFv**

**Untreated**

**3D8 scFv**

**H1N1+H3N2**

**10^-6^**

**OC43+PEDV**

**10^-3^**

**Untreated**

**3D8 scFv**

**E**

**Untreated**

**3D8 scFv**

**Adapted-H1N1+OC43**

**10^-6^**

**10^-6^**

**Oseltamivir**

**10^-5^**

**Untreated**

**3D8 scFv**

**10^-4^**

**10^-3^**

**Chloroquine**

**10^-3^**

**OC43**

**Adapted-H1N1**

**Fig S3. Plaque reduction assay**. The supernatants from the 3D8-treated cells were harvested, and a plaque reduction assay was performed to observe the infectious viral titer. The plaques were counted as shown in the graph in single infection of (A) coronaviruses hCoV-OC43, PEDV, (B) influenza viruses H1N1/PR8, H3N2/X-31, and adapted-H1N1. And in co-infection of (C) coronaviruses (hCoV-OC43 and PEDV), (D) IAVs (H1N1/PR8 and H3N2/X3-1), (E) IAV and coronavirus (adapted-H1N1 and hCoV-OC43).

**Polyclonal rabbit anti-GAPDH antibody**

**Polyclonal rabbit anti-GAPDH antibody**

**Monoclonal antibody to hCoV-OC43 nucleoprotein**

**Monoclonal antibody to PEDV**

**Polyclonal rabbit anti-HA antibody**

**Polyclonal rabbit anti-HA antibody**

**Fig S4. Original photos of Western blotting method.** These are the uncropped photos of all the Western blotting’s results that were used in the manuscript with the full-length membranes (with the full ladder size from 17 kDa to 130 kDa). The samples were treated with different primary antibodies. After that, the membranes were incubated with the appropriate secondary antibodies. Finally, membranes were incubated with the enhanced chemiluminescence solution, and then exposed to the films. Finally, once the films were developed, they were scanned with a CanoScan 5600F scanner.

Note: We exposed the membranes and developed X-ray films in dark room which were not able to show the edge of membranes on the films (since the exposure time was not too long to burn the films to observe all the edges of membranes).

These lanes which are bordered by Red, Green, Blue, Purple border boxes are represented for each Fig 1, 2, 3, 4, respectively.
